# Exploring the Boundaries of Cyclometalated Iridium(III) Sensitizers in Photoelectrochemical Organic Transformations

**DOI:** 10.1021/acsami.5c20212

**Published:** 2025-12-12

**Authors:** Andrea Mantovani, Annagioia Mastrolorenzo, Edoardo Marchini, Paola Manini, Mirco Natali

**Affiliations:** † Department of Chemical, Pharmaceutical and Agricultural Sciences (DOCPAS), 9299 University of Ferrara, Via L. Borsari 46, Ferrara 44121, Italy; ‡ Department of Chemical Sciences, 9307 University of Naples Federico II, Via Cintia 4, Naples 80126, Italy

**Keywords:** iridium(III) complexes, DSPEC, benzyl alcohol, radical cation Diels−Alder, value-added organics, photoelectrochemistry, oxidation

## Abstract

Dye-sensitized photoelectrochemical cells (DSPECs) are currently at the forefront of solar-to-chemical energy conversion technologies. Although water oxidation to dioxygen has long been the preferred reaction at the photoanodic compartment, recent research has increasingly focused on oxidation processes for the synthesis of value-added organic compounds. Quite surprisingly, within this framework, cyclometalated iridium­(III) complexes have received negligible attention as photoactive components in DSPEC photoanodes, in spite of their intriguing photophysical and electrochemical properties. With the aim of filling this gap, this work explores the application of two iridium­(III) complexes (**Ir1** and **Ir2**), differing in the presence of fluorinated substituents, as light-harvesting sensitizers anchored onto mesoporous TiO_2_ photoelectrodes. These systems were employed to drive two relevant oxidation processes: the TEMPO-mediated oxidation of benzyl alcohol (BzOH) to benzaldehyde and the radical cation Diels–Alder reaction between *trans*-anethole (TA) and isoprene (ISO). In the oxidation of BzOH to benzaldehyde, maximum photocurrent densities on the order of 0.5–0.7 mA·cm^–2^ were recorded, but the photoelectrodes proved substantially inefficient (APCE between 2.2% and 2.4%). Under operative conditions, low Faradaic efficiencies (FEs) for benzaldehyde formation were also registered (42% and 32% for **Ir1** and **Ir2**, respectively), associated with a rapid decrease in photocurrent densities, particularly in the case of the fluorinated complex. In contrast, the DSPEC system operating without a redox mediator exhibits markedly improved performances (photocurrent densities on the order of 0.7 mA·cm^–2^, APCE up to 19%), with quantitative conversion of the TA substrate under bulk electrolysis conditions. Interestingly, for this latter reaction, the enhanced oxidative power of the fluorinated sensitizer contributes to the increased reactivity. A combination of photoelectrochemical and transient absorption spectroscopy studies has been performed to rationalize the observed behavior. The results highlight how the molecular design and electronic properties of the dye component in DSPECs should be rationally engineered to align with the thermodynamic and kinetic requirements of the targeted chemical transformation.

## Graphical abstract

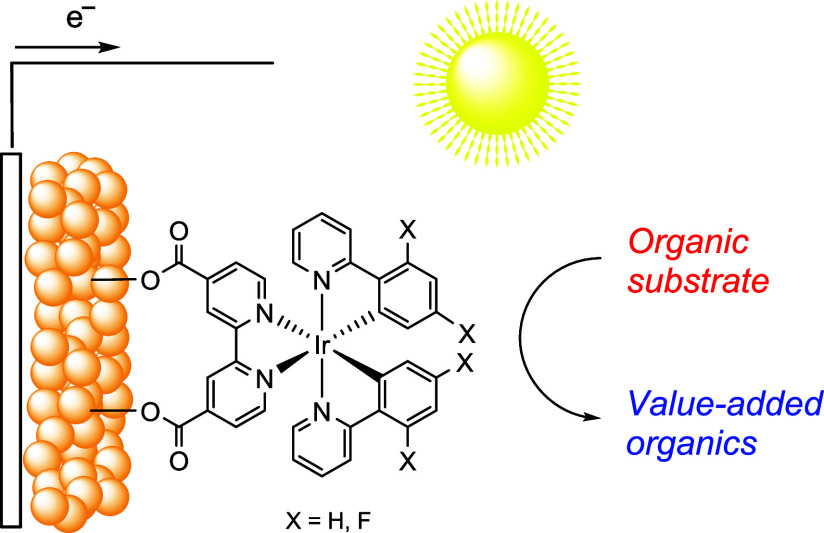

## Introduction

Harnessing sunlight to produce value-added molecules represents a promising approach toward sustainable development. In this context, dye-sensitized photoelectrochemical cells (DSPECs) have emerged as a flexible and effective platform.^1–3^ In these systems, a molecular dye absorbs visible light and injects an electron into a wide-bandgap semiconductor. The resulting oxidized dye can then mediate the oxidation of the substrate at the photoanode surface, while the injected electron is transferred to a cathode, often reducing water to dihydrogen.

Traditionally, DSPEC systems have focused on water splitting, with the oxygen evolution reaction (OER) taken as a benchmark process within the photoanodic compartment.^4–8^ However, the high overpotential, sluggish kinetics, and limited economic value of the oxygen product have prompted a paradigm shift toward more valuable and kinetically favorable oxidation reactions, specifically, the oxidation of organic substrates.^9^ This shift from water to organic oxidation offers several advantages indeed. First, many such reactions are thermodynamically and kinetically more favorable than water oxidation, enabling higher photocurrents and improved energy efficiency. Furthermore, the oxidation of organic substrates can yield high-value products and fine chemicals, adding economic incentive to solar-driven PEC technologies.

Within this framework, benzyl alcohol has been commonly considered as a prototypical substrate for evaluating DSPEC performance due to its well-understood oxidation pathway and relevance in industrial contexts. Its selective conversion to benzaldehyde under visible light irradiation indeed provides a model reaction for probing the interplay between dye structure and semiconductor properties.^9–14^ This approach has been also extended to a broader range of alcohols,^15,16^ other organic substrates,^17^ and more challenging chemical transformations (e.g., C–H bond activation),^18^ opening new avenues for solar-assisted organic synthesis and biomass valorization. More recently, Schanze and coworkers demonstrated the use of a sensitized TiO_2_ photoanode in a DSPEC to drive a radical cation Diels–Alder reaction under visible light, highlighting the potential of these devices for enabling organic transformations through single-electron oxidation pathways.^19^

Regardless of the nature of the target chemical transformation, at the heart of DSPEC lies the sensitizer, i.e., the molecular species that captures visible light and initiates the charge separation process. In this respect, ruthenium polypyridine complexes have received substantial attention thanks to their suitable optical and electrochemical properties.^20^ Porphyrins and organic sensitizers have also been profitably exploited to expand the light-harvesting capability toward the red portion of the visible spectrum or to introduce new mechanistic scenarios.^14,18^ Interestingly, while most studies have focused on the mere application and optimization of a certain sensitizer toward the target transformation, less is known about the critical role of the dye and its potential, general employment toward diverse chemical oxidation reactions. Starting from this background, we report herein the use of two cyclometalated iridium­(III) complexes (**Ir1** and **Ir2**) to promote the TEMPO-mediated oxidation of benzyl alcohol (BzOH) to benzaldehyde and to drive the radical cation Diels–Alder reaction between *trans*-anethole (**TA**) and isoprene (**ISO**, Scheme 1).^19^ Whereas iridium complexes have already been reported as effective sensitizers in n-type dye-sensitized solar cells (DSSCs),^21–29^ to the best of our knowledge, no reports to date have explored their application in DSPECs for organic oxidation reactions. This gap also presents a compelling opportunity to investigate the potential of this class of sensitizers in DSPEC systems, particularly given their favorable photophysical and electrochemical properties. We will show that the ability of iridium complexes to function as effective sensitizers strictly depends on the nature of the organic transformation. Furthermore, we demonstrate that increasing the oxidation power of the dye with the introduction of fluorinated groups (from **Ir1** to **Ir2**), in principal effective to activate more inert substrates based on simple thermodynamic arguments, turns out to be beneficial only in the Diels–Alder reaction, whereas it becomes disadvantageous in the presence of a redox mediator due to the enhancement of self-degradation phenomena.

**Scheme 1:**
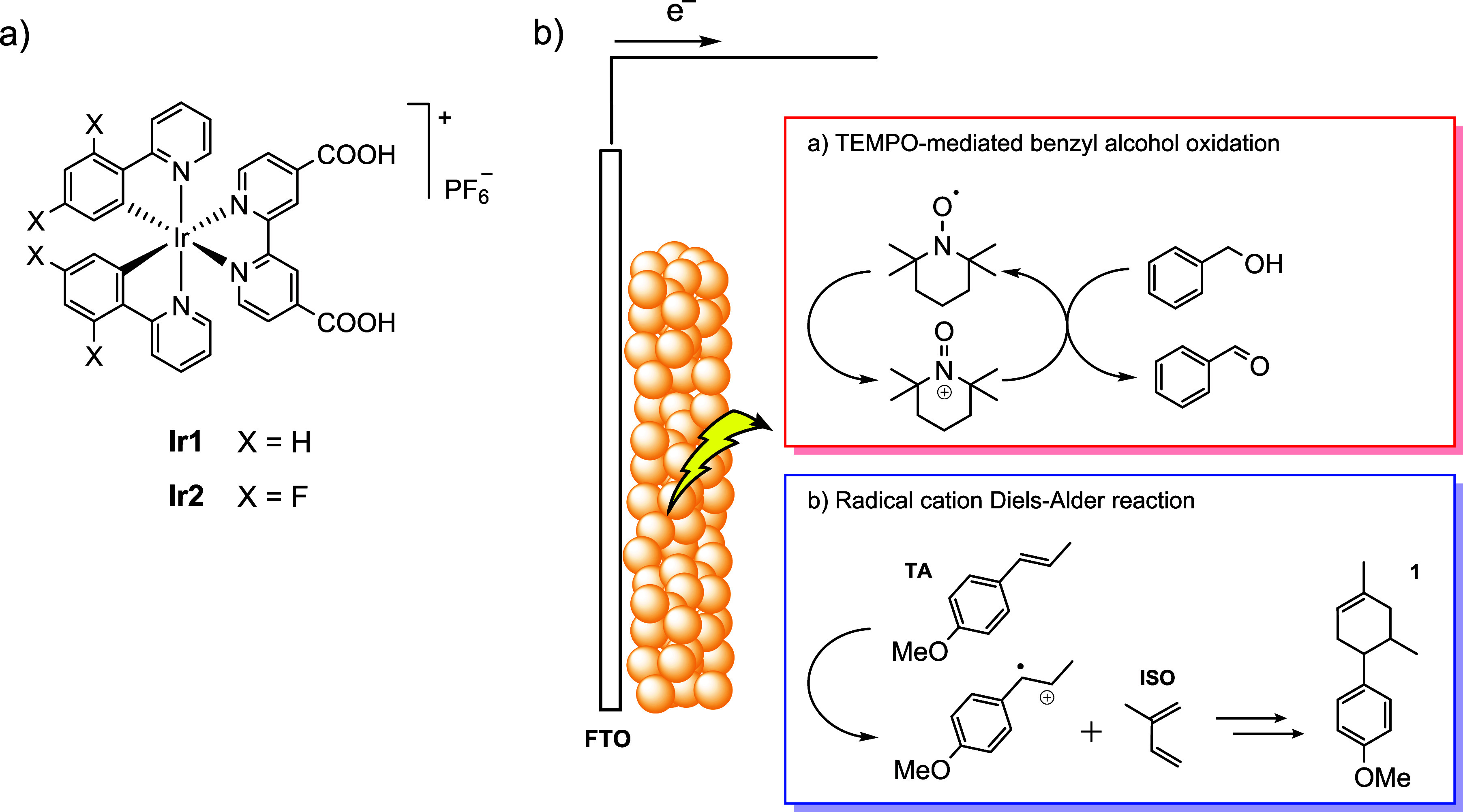
a) Molecular Structure of Complexes **Ir1** and **Ir2** and b) Schematic Representation of the Dye-Sensitized TiO_2_ Photoanodes and the Target Photochemical Reactions Explored in the Present Work

## Experimental Section

### Materials and Methods

All reagents were obtained from standard suppliers and used without further purification. An electrochemical grade acetonitrile solvent was employed. Fluorine-doped tin oxide (FTO) substrates were purchased from Pilkington and subjected to a rigorous cleaning protocol to ensure optimal surface conditions for electrode fabrication. The cleaning process involved sequential ultrasonication for 10 min each in a 2% w/w aqueous Alconox solution and 2-propanol. After drying at room temperature, the slides were thermally treated in air at 450 °C to remove residual organic contaminants. The substrates were then allowed to cool down. Cleaned FTO slides were used immediately following this procedure to minimize surface recontamination and ensure reproducibility in subsequent fabrication steps.

^1^H, ^19^F, and ^13^C NMR were registered on a Bruker DRX (400 MHz) instrument. Chemical shifts are given in ppm relative to the NMR standard tetramethylsilane (TMS), and J values are given in Hz. ^1^H,^1^HCOSY, ^1^H,^13^C HSQC, and ^1^H,^13^C HMBC experiments were run at 400 MHz using standard pulse programs. MALDI mass spectra were recorded on an AB Sciex TOF/TOF 5800 instrument using 2,5-dihydroxybenzoic acid as the matrix. Spectra represent the sum of 15000 laser pulses from randomly chosen spots per sample position. Analytical and preparative TLC were performed on silica gel plates F254 (0.25 and 0.5 mm, respectively) and were visualized using a UV lamp (λ = 254 nm) and a fluorescence lamp (λ = 356 nm).

Photocurrent density–voltage curves (JV) and bulk electrolysis measurements were performed using an Autolab PGSTAT 302 N potentiostat in a three-electrode configuration using a saturated calomel electrode (SCE) as the reference and a Pt counter electrode. The cell was irradiated with an Abet solar simulator equipped with an AM1.5G filter adjusting the spectral irradiance to 1 sun (0.1 W·cm^–2^) by means of a Newport 1918-C Power Meter. Incident photon-to-current conversion efficiency (IPCE) was measured in a three-electrode configuration under the monochromatic illumination generated by an air-cooled Luxtel 175 W Xe lamp coupled to an Applied Photophysics monochromator. Incident irradiance was measured with a calibrated silicon photodiode. In all photoelectrochemical experiments, irradiation was provided from the back side of the photoelectrode. Photoelectrochemical experiments were repeated multiple times to ensure reproducibility of the data, and the results shown are representative of the consistent behavior observed across independently prepared photoanodes. Absorption spectra were recorded at room temperature using either a Jasco V-560 or a Jasco V-570 spectrophotometer. Luminescence spectra were recorded using either an Edinburgh Instrument or a Jasco FP-750 spectrofluorometer. Luminescence quantum yields (Φ) in solution were calculated using [Ru­(bpy)_3_]^2+^ as reference (Φ = 0.062 in acetonitrile solution). Time-resolved luminescence decays and transient absorption spectroscopy measurements were conducted using a custom laser spectrometer comprising a Continuum Surelite II Nd:YAG laser (fwhm = 8 ns) with frequency tripled option (355 nm). Photomultiplier signals (kinetic traces) were processed using a Teledyne LeCroy 604Zi (400 MHz, 20 GS/s) digital oscilloscope.

### Synthesis and Characterization

#### Synthesis of [Ir­(ppy)_2_Cl]_2_ (**1**)

Ppy (71.4 μL, 0.5 mmol) and IrCl_3_·H_2_O (59.7 mg, 0.2 mmol) were added to a 3:1 (v/v) mixture of EtOCH_2_CH_2_OH (9 mL) and H_2_O (3 mL). The mixture was purged with argon and heated to reflux (120 °C) for 24 h (reaction completion checked via TLC; CHCl_3_/MeOH 9:1). Upon cooling to room temperature, the suspension was concentrated under vacuum and completely dried to give the crude dinuclear complex **1** as a yellow powder (59 mg, 55%). The latter was used without further purification for the next reaction steps. ^1^H-NMR (400 MHz, CDCl_3_) δ ppm: 9.25 (d, *J* = 5.5 Hz, 4H), 7.97 (d, *J* = 7.8 Hz, 4H), 7.82 (td, *J* = 7.9, 1.2 Hz, 4H), 7.56 (dd, *J* = 7.9, 1.2 Hz, 4H), 6.90–6.78 (m, 8H), 6.63 (td, *J* = 7.9, 1.2 Hz, 4H), 5.89 (dd, *J* = 7.9, 1.2 4H).

#### Synthesis of Ir­(ppy)_2_H_2_dcbpy (**Ir1**)

**1** (30 mg, 0.028 mmol) and H_2_dcbpy (15.3 mg, 0.062 mmol) were added to a 2:1 v/v mixture of EtOH (4 mL) and H_2_O (2 mL). The reaction mixture was heated to reflux (90 °C) for 5 h. Upon cooling to room temperature, NH_4_PF_6_ (20 equiv) was added. After stirring for 30 min at r.t., the solvent was removed under reduced pressure, and the crude mixture was extracted with MeOH, allowing the removal of unreacted H_2_dcbpy as a white precipitate (cycles of resuspension in methanol and centrifugation). The combined methanolic phases were then concentrated under vacuum, and the crude mixture was washed with Et_2_O leading to the spectroscopically pure product **Ir1** as a light-red powder (38.5 mg, 77%). ^1^H-NMR (400 MHz, DMSO-d_6_) δ ppm: 9.24 (s, 2H), 8.28 (d, *J* = 8.2 Hz, 2H), 8.09 (d, *J* = 5.7 Hz, 2H), 8.04 (d, *J* = 5.7 Hz, 2H), 7.95 (m, 4H), 7.67 (d, *J* = 5.7 Hz, 2H), 7.14 (t, *J* = 6.6 Hz, 2H), 7.04 (t, *J* = 7.4 Hz, 2H), 6.92 (t, *J* = 7.4 Hz, 2H), 6.17 (d, *J* = 7.5 Hz, 2H), 12.26 (s, 2H). ^13^C-NMR (100 MHz DMSO-d_6_) δ ppm: 172.1, 171.2, 161.0, 160.5, 155.9, 154.8, 153.8, 143.5, 136.3, 135.1, 132.4, 129.9, 128.7, 128.0, 127.2, 125.4, 124.8. ^19^F-NMR (376 MHz, DMSO-d_6_) δ ppm: −70.82 (d, *J =* 710.6 Hz). MALDI^+^ MS: [(M^+^ – 2PF_6_)] *m/z* 745.2.

#### Synthesis of [Ir­(dfppy)_2_Cl]_2_ (**2**)

Dfppy (72.2 μL, 0.5 mmol) and IrCl_3_·H_2_O (59.7 mg, 0.2 mmol) were added to a 3:1 (v/v) mixture of EtOCH_2_CH_2_OH (9 mL) and H_2_O (3 mL). The mixture was purged with argon and heated to reflux (120 °C) for 24 h (reaction completion checked via TLC; CHCl_3_/MeOH 9:1). Upon cooling to room temperature, the suspension was concentrated under vacuum and completely dried to give the crude dinuclear complex **2** as a dark-yellow powder (65 mg, 53%). The latter was used without further purification for the next reaction steps. ^1^H-NMR (400 MHz, DMSO-d_6_) δ ppm: 9.51 (dd, *J =* 6.0, 1.0 Hz, 2H), 9.29 (dd, *J =* 5.9, 1.0 Hz, 2H), 8.04 (d, *J =* 7.9 Hz, 2H), 7.99 (d, *J =* 7.6 Hz, 2H), 7.93 (m, 2H), 7.85 (m, 2H), 7.40 (ddd, *J =* 7.73, 6.0, 1.4 Hz, 2H), 7.31 (ddd, *J =* 7.4, 5.9, 1.4 Hz, 2H), 6.55 (m, 4H), 5.47 (m, 2H), 4.80 (dd, *J =* 8.8, 2.5 Hz, 2H). ^19^F-NMR (376 MHz, DMSO-d_6_) δ ppm: −108.41 (d, *J =* 9.4 Hz), −110.60 (d, *J =* 9.1 Hz).

#### Synthesis of Ir­(dfppy)_2_H_2_dcbpy (**Ir2**)

**2** (30 mg, 0.026 mmol) and H_2_dcbpy (14.3 mg, 0.059 mmol) were added to a 2:1 v/v mixture of EtOH (4 mL) and H_2_O (2 mL). The reaction mixture was heated to reflux (90 °C) for 5 h. Upon cooling to room temperature, NH_4_PF_6_ (20 equiv) was added. After stirring for 30 min at r.t., the solvent was removed under reduced pressure, and the crude mixture was extracted with MeOH, allowing the removal of unreacted H_2_dcbpy as a white precipitate (cycles of resuspension in methanol and centrifugation). The combined methanolic phases were then concentrated under vacuum, and the crude mixture was washed with Et_2_O, leading to the pure product **Ir2** as a bright-yellow powder (43.7 mg, 87%). ^1^H-NMR (400 MHz, DMSO-d_6_) δ ppm: 9.29 (s, 2H), 8.31 (d, *J* = 8.6 Hz, 2H), 8.09–8.02 (m, 6H), 7.75 (d, *J* = 5.5 Hz, 2H), 7.22 (t, *J* = 6.6 Hz, 2H), 7.00 (d, *J* = 9.9 Hz, 2H), 5.60 (dd, *J* = 8.2 Hz, 2H), 12.52 (s, 2H). ^13^C-NMR (100 MHz, DMSO-d_6_) δ ppm: 168.5, 166.6, 164.5, 164.4, 162.4, 155.2, 147.8, 147.6, 142.3, 138.6, 129.4, 127.9, 126.5, 125.1, 124.1, 115.7, 115.6. ^19^F-NMR (376 MHz, DMSO-d_6_) δ ppm: −108.6 (d, *J =* 10.4 Hz), −106.5 (d, *J =* 10.4 Hz), −70.13 (d, *J =* 711.3 Hz). MALDI^+^ MS: [(M^+^ – 2PF_6_^–^)] *m/z* 817.2.

#### Electrode Preparation

TiO_2_ electrodes were fabricated following a previously reported protocol.^30^ Initially, a compact blocking layer was deposited by spin-coating onto FTO glasses (10 s at 1000 rpm, 2 s at 2000 rpm) of a 0.3 M solution of titanium­(IV) tetra­(isopropoxide) in 1-butanol followed by heating at 500 °C for 15 min. Then, a layer of TiO_2_ paste (Solaronix) was applied via doctor-blading and subjected to thermal treatment in a muffle under the following temperature profile: ramp from room temperature to 120 °C at 10 °C/min, followed by a ramp to 450 °C at 11 °C/min, with a dwell time of 30 min at 450 °C. Subsequently, the temperature was increased to 500 °C at 5 °C/min and held for 10 min. This deposition of TiO_2_ paste and annealing process was repeated three times to ensure uniformity and optimal film thickness (“triple layer” electrodes). The resulting thickness is ca. 15 μm, as determined using a profilometer (Alpha-Step D500, KLA). For electrodes named “triple layer + scattering layer”, an additional TiO_2_ scattering paste (Solaronix) was deposited onto the triple layer using the same blading technique, followed by annealing under identical thermal conditions. A post-treatment was performed by casting a 0.4 M TiCl_4_ solution onto the surface of the active substrate, followed by overnight hydrolysis at room temperature in a closed chamber. Subsequently, the electrodes were washed with distilled water and heated in a muffle at 450 °C for 30 min. The resulting thickness is ca. 17 μm, as determined via profilometry.

ZrO_2_ electrodes were fabricated on FTO substrates via blade-casting of a nanocrystalline ZrO_2_ paste, followed by thermal treatment.^31^ The films were dried and subsequently sintered at 450 °C for 45 min to ensure structural consolidation. The aqueous paste, containing 15% w/w ZrO_2_, was synthesized through controlled acidic hydrolysis of zirconium­(IV) tetra­(isopropoxide). This was followed by hydrothermal treatment at 220 °C for 12 h to promote nucleation and crystal growth. To enhance sintering, Carbowax 20000 was added at a concentration of 40% w/w relative to ZrO_2_.

TiO_2_ and ZrO_2_ electrodes were sensitized by overnight soaking in 15 mL acetonitrile solution containing 0.5 mM **Ir1** or **Ir2**. Subsequently, the electrodes were rinsed with acetonitrile and dried under airflow.

## Results

### Synthesis and Characterization

The iridium complexes **Ir1** and **Ir2** were prepared following an easy two-step procedure (Scheme 2). In the first step, following a reported procedure with slight modifications,^32^ iridium­(III) chloride was reacted with the proper cyclometalating ligand, 2-phenylpyridine (ppy) or 2-(2’,4’-difluorophenyl)­pyridine (dfppy), in a mixture of 2-ethoxyethanol and water under reflux conditions and an argon atmosphere. After 24 h, the dinuclear chloro-bridged iridium complexes **1** and **2** were obtained as yellow solids in good yields. The identity of the complexes was confirmed by NMR analysis.^33,34^

**Scheme 2:**
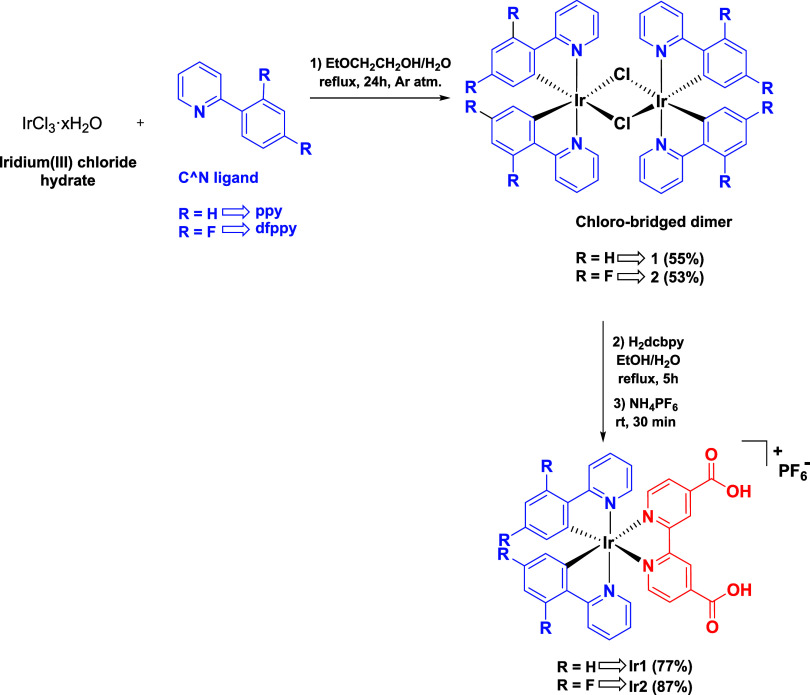
Schematic Procedure for the Synthesis of Iridium Complexes **Ir1** and **Ir2**

In the second step, the insertion of the 4,4’-dicarboxy-2,2’-bipyridine (H_2_dcbpy) was pursued by treating the ancillary ligand with the complexes **1** and **2** in an EtOH/H_2_O solution under reflux conditions. After 5 h, the reaction mixtures were treated with an excess of NH_4_PF_6_ to promote the precipitation of the two complexes **Ir1** and **Ir2**, collected in good yields as light-red and light-yellow solids, respectively. The solids were washed extensively with methanol to remove the unreacted H_2_dcbpy ligand and subjected to crystallization with diethyl ether for further purification. The identity of the complexes **Ir1** and **Ir2** was confirmed by 1D and 2D NMR analyses and mass spectrometry.^35–37^

The absorption and emission properties of the **Ir1** and **Ir2** complexes in solution were investigated by registering UV–vis and emission spectra in diluted acetonitrile (Figure S1). All the results are reported in Table S1. In good agreement with literature data,^38,39^ the iridium complexes **Ir1** and **Ir2** exhibit intense absorption bands between 240 and 320 nm with high absorption coefficients (>10000 M^–1^ cm^–1^), which can be assigned to spin-allowed ligand-centered transitions ^1^LC (^1^π-π*) localized on the H_2_dcbpy and C^N ligands. The absorptions between 320 and 410 nm are attributed to spin-allowed singlet-to-singlet metal-to-ligand charge-transfer (^1^MLCT) and ligand-to-ligand charge-transfer (^1^LLCT), which are common among iridium compounds. In addition, weaker absorptions above 410 nm are likely to include singlet-to-triplet LLCT and MLCT transitions of spin-forbidden character due to the enhanced spin–orbit coupling of the iridium center. The presence of the fluorine atoms in the **Ir2** complex is responsible for a blue shift of the absorption maxima because of the stabilization of the HOMO and the increase of the HOMO–LUMO gap.

According to TD-DFT calculations,^40^ the HOMO of both **Ir1** and **Ir2** is mainly located on the metal center and the phenyl moiety of C^N ligands, while the LUMO lies primarily on the N^N ligand (H_2_dcbpy). However, the presence of the electron-withdrawing −COOH groups on the H_2_dcbpy ligand lowers the energy of the LUMO, thereby decreasing the energy gap between the HOMO and the LUMO. This facilitates electron transitions that involve multiple orbitals, including HOMO or HOMO–1 to LUMO or some orbitals above.

The iridium complexes exhibit emission profiles associated with a ^3^MLCT phosphorescence and featuring high luminescence yields (Table S1). Also in this case, the introduction of fluorine atoms in **Ir2** results in a blue-shifted maximum with respect to **Ir1**. Moreover, a significant increase in the emission quantum yield was evident when passing from **Ir1** (Φ = 3.4%) to **Ir2** (Φ = 30.5%), mainly as a result of energy-gap law considerations. The emission lifetimes are 42 and 810 ns for **Ir1** and **Ir2**, respectively, in agreement with previous reports.^41^

### Optical and Electrochemical Properties on Thin Film

The spectroscopic and electrochemical properties of the cyclometalated iridium complexes **Ir1** and **Ir2** were then examined on ZrO_2_ thin films. This substrate indeed represents an environment that closely mimics the operational conditions of our iridium complexes in the DSPEC system.

Figure 1a depicts the absorption and emission spectra of complexes **Ir1** and **Ir2**, while Figure 1b displays the cyclic voltammetry (CV) recorded in acetonitrile (0.1 M LiClO_4_). Both the absorption and the luminescence properties qualitatively resemble those observed in solution conditions (see above), only differing in an appreciable blue-shift of the emission bands resulting from the binding of the carboxylic acid functional groups to the oxide support (see Table S1). Under cathodic scan, both complexes **Ir1** and **Ir2** show an irreversible reduction process occurring at comparable potentials (peak potentials of *E* = −1.36 and −1.28 V vs SCE, respectively), which is assigned to the reduction of the ancillary diimine ligand.^40^ Under anodic scan, an irreversible process is observed, attributable to oxidation of the iridium center Ir­(III)/Ir­(IV), whose potential differs depending on the type of metal complex considered (peak potentials of *E* = +1.42 and +1.68 V vs SCE for **Ir1** and **Ir2**, respectively). The more positive potential exhibited by **Ir2** is consistent with the electron-withdrawing character of the fluorine substituents in the cyclometalated ligands.^42,43^ According to these findings, the oxidized complex **Ir2** is expected to be a stronger oxidant than the respective **Ir1** complex. Importantly, comparison of the potential values with those of the target substrates (i.e., TEMPO for BzOH oxidation and **TA** for the Diels–Alder reaction, see Table S2) clearly establishes the thermodynamic feasibility of the planned electron transfer processes at the semiconductor-electrolyte interface.

**Figure 1:**
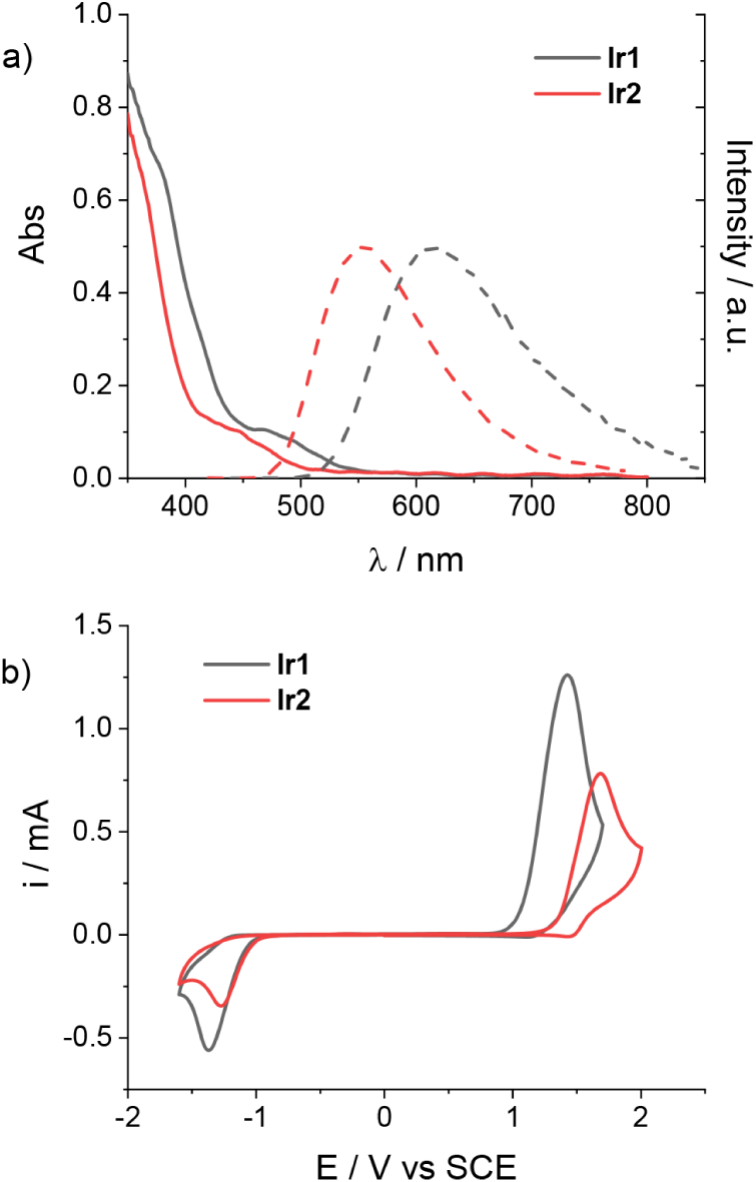
a) Normalized absorption (solid lines) and emission spectra (dashed lines) of **Ir1** and **Ir2** adsorbed on ZrO_2_ thin films and b) CV of **Ir1** and **Ir2** on ZrO_2_ in acetonitrile solution with 0.1 M LiClO_4_ as the supporting electrolyte.

The spectroscopic energies (*E* ^00^) of the triplet state of both complexes **Ir1** and **Ir2** were then extracted from the intersection between the normalized absorption and emission bands, providing values of 2.30 and 2.49 eV for **Ir1** and **Ir2**, respectively, nicely reflecting the HOMO–LUMO energy gaps inferred from CV analysis. Using these values, the reduction potentials of the triplet excited state can be determined (Table S2), pointing to the thermodynamic ability of both complexes to undergo electron injection from the triplet state into the TiO_2_ conduction band. We should also consider that, akin to ruthenium polypyridine complexes,^44,45^ electron injection might also occur from the higher-lying singlet excited state,^41^ from which the driving force for the process is expected to be even larger by a factor of ca. 0.2 eV.

### Photoelectrochemical Tests in the Presence of a Sacrificial Hole Scavenger

Before investigating the target transformations of organic substrates, we examined the photoelectrochemical performances of TiO_2_ photoelectrodes sensitized with both **Ir1** and **Ir2** toward the oxidation of LiI as a sacrificial hole scavenger in acetonitrile solution (0.1 M LiClO_4_). A triple TiO_2_ layer was used for these measurements. As depicted in Figure 2a, measurable photocurrent densities are detected starting at onset potentials of ca. −0.35 V vs SCE in both cases and reaching values at plateau on the order of 2.5 and 1.6 mA·cm^–2^ for **Ir1** and **Ir2**, respectively.

**Figure 2:**
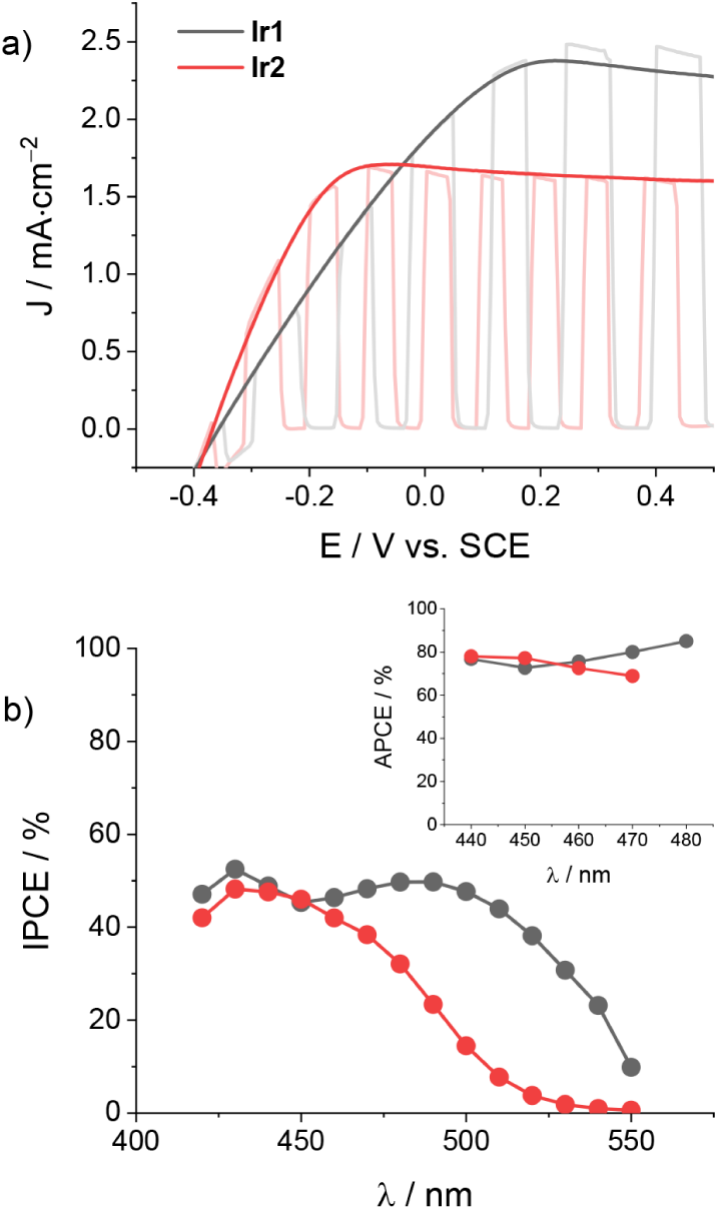
a) JV curves of TiO_2_-sensitized electrodes (triple layer) with **Ir1** and **Ir2** under direct or chopped irradiation (1 sun, cutoff filter at 395 nm) and b) the corresponding IPCE spectra and APCE spectra (inset) at +0.2 V vs SCE in the presence of 0.1 M LiI and 0.1 M LiClO_4_ in acetonitrile.

We next turn to the measurement of the photoaction spectra in order to extract the efficiency of the charge injection of the iridium complexes onto the TiO_2_ conduction band under experimental conditions relevant to the target organic transformations. Indeed, after the efficient regeneration of Ir­(III) by I^–^, the slow recombination kinetics between the photoinjected electrons and the generated I_3_^–^ species ensure a charge collection efficiency (η_cc_) close to unity, with the photocurrent essentially depending on the charge injection yield (η_inj_) and the light harvesting efficiency (LHE).^14^ The IPCE spectra of both TiO_2_ electrodes are displayed in Figure 2b and show maximum values on the order of 53% and 49% in the case of TiO_2_ electrodes sensitized with **Ir1** and **Ir2**, respectively. Normalization of these spectra by the LHE leads to the estimation of the absorbed photon-to-current conversion efficiency (APCE), namely the internal quantum efficiency of the device, obtaining mean values of 78% and 74% (see inset in Figure 2b) for **Ir1** and **Ir2**, respectively. In agreement with the previous assumption, these quantities confirm efficient charge injection of the photoexcited iridium complexes into the TiO_2_ conduction band. In this respect, the slightly lower value measured in the case of the fluorinated **Ir2** complex with respect to the unsubstituted **Ir1** is consistent with the lower driving force for charge injection, as predicted based on electrochemical data (Table S2).

### TEMPO-Mediated Benzyl Alcohol Oxidation

Once established the ability of both **Ir1** and **Ir2** complexes to effectively sensitize TiO_2_, we next examined the activity of the resulting photoelectrodes to promote the TEMPO-mediated oxidation of BzOH to benzaldehyde. Before entering reactivity studies, we performed a screening of the experimental variables in order to identify optimized conditions. We first started by employing a triple TiO_2_ layer and identified a concentration of 10 mM TEMPO in acetonitrile (0.1 M LiClO_4_) as the optimum one to achieve the largest photocurrent densities. In this regard, the progressive loss of activity at larger TEMPO loadings (Figure S4) stems from the competition in light-absorption by the redox mediator, clearly hampering effective light-harvesting by the iridium sensitizers.^14^ As a second step, since the TEMPO-mediated catalysis requires the use of a base, we investigated the role and effect of different bases (Figure S5) and observed that the largest photocurrent densities can be achieved using lithium bis­(trifluoromethanesulfonylimide) (LiTFSI), acting both as a supporting electrolyte and as a base. Finally, to improve the light-harvesting capability, we examined the influence of an additional TiO_2_ top scattering layer (particle size >100 nm) and detected improved photocurrent densities over the transparent counterpart.

The JV curves recorded under optimized conditions (triple TiO_2_ layer with scattering layer, 10 mM TEMPO, 0.1 M LiTFSI in acetonitrile) in the presence of 50 mM BzOH are reported in Figure 3a. For **Ir1** and **Ir2**, detectable photocurrent densities can be measured with onsets of −0.2 and −0.3 V, respectively, which plateau at higher potential values reaching maximum photocurrent densities of 0.67 and 0.51 mA·cm^–2^, respectively. These values are substantially larger than those measured using photoanodes based on ruthenium sensitizers^15^ as well as on organic dyes such as perylenes and polyquinoids,^12,13^ whereas they are lower than those recently recorded using perfluorinated porphyrin sensitizers^14^ (see Table S3). A detailed comparison of the JV curves for complexes **Ir1** and **Ir2** shows a more negative onset potential and a more squared profile in the case of **Ir2,** together with the presence of more pronounced spikes in the chopped scan of **Ir1**. All these findings suggest the presence of some recombination phenomena involving the oxidized TEMPO mediator and the injected electron, which are stronger in **Ir1**-sensitized TiO_2_ electrodes than **Ir2**-based ones.

**Figure 3:**
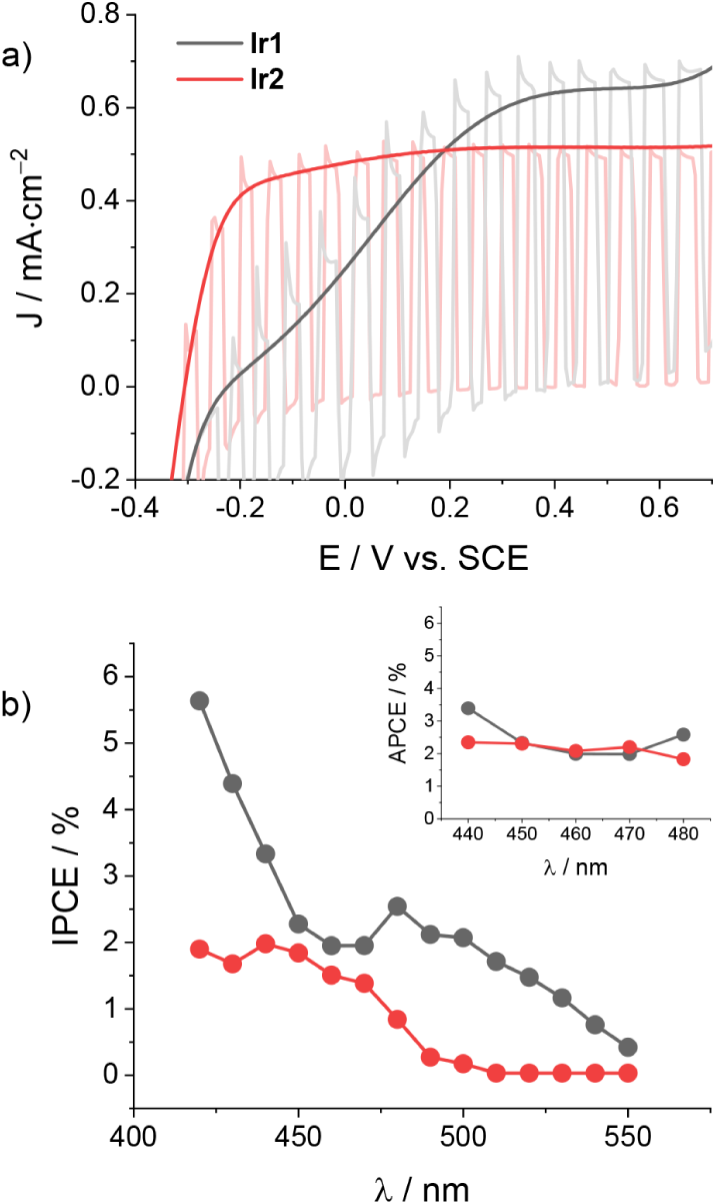
a) JV curves of TiO_2_-sensitized electrodes (triple layer + scattering layer) with **Ir1** and **Ir2** under direct or chopped irradiation (1 sun, cutoff filter at 395 nm) and b) the corresponding IPCE spectra and APCE spectra (inset) at +0.5 V vs SCE in the presence of 10 mM TEMPO, 50 mM BzOH, and 0.1 M LiTFSI in acetonitrile.

Figure 3b exhibits the IPCE spectra of both TiO_2_ electrodes measured under optimized conditions. The spectral profiles of the photoaction spectra follow the shape of the absorption spectra (Figure 1a), confirming that in both cases the measured photocurrents originate from TiO_2_ sensitization by the iridium complexes **Ir1** and **Ir2**. A maximum IPCE value of 5.5% is recorded for complex **Ir1** at 420 nm, likely including some contribution from the bare TiO_2_ electrode, whereas a value of 2.5% is measured in the triplet MLCT transition at 480 nm. Slightly lower IPCE values are measured for complex **Ir2** (1.9% at 420 and 440 nm), in fairly good agreement with the larger photocurrent densities measured at plateau for **Ir1**-sensitized electrodes than **Ir2**-based ones (Figure 3a).

Normalization of the IPCE spectra by the LHE allowed for the extraction of the resulting APCE. The values (inset in Figure 3b) are appreciably constant in the main absorption bands. Average values of 2.4% and 2.2% can be extracted for complexes **Ir1** and **Ir2**, respectively, indicating that the higher IPCE in the case of **Ir1** partly stems from a larger LHE. The values here recorded are within the same order of those registered for TiO_2_ electrodes sensitized with a porphyrin-TEMPO dyad,^16^ while they are consistently lower than those reported for TEMPO-mediated BzOH oxidation using perfluorinated porphyrin sensitizers,^14^ possibly suggesting enhanced recombination routes in the case of iridium-based dyes **Ir1** and **Ir2** with respect to the latter. These phenomena are feasible for fast and reversible redox couples, probably enhanced by the lower FTO passivation.

Bulk electrolysis experiments (acetonitrile solution with 10 mM TEMPO, 0.1 M LiTFSI, and 50 mM BzOH) were finally performed in a two-compartment cell upon application of a constant bias of +0.5 V vs SCE in order to estimate the performances of the two electrodes toward the generation of benzaldehyde. The corresponding Faradaic efficiency (FE) was determined after 6 h in the case of **Ir1**-sensitized TiO_2_ electrodes and 2 h for **Ir2**-sensitized electrodes, i.e., when substantial abatement of the photocurrent density was observed (Figure S6). FEs of 42% and 32% (Figures S7 and S8) were recorded for **Ir1** and **Ir2**, respectively, indicating the occurrence of parallel deactivation pathways under operative conditions. These results are consistent with the decaying profile of the chronoamperometry traces (Figure S6).

### Radical Cation Diels–Alder Reaction

Schanze and coworkers recently reported the possibility of promoting a Diels–Alder reaction between **TA** and **ISO** mediated by the radical cation of the former produced via photoelectrochemical means on a TiO_2_ electrode sensitized with a ruthenium polypyridine complex.^19^ While successful, the efficiency of the system was mainly hampered by the slow kinetics of the hole transfer from the oxidized sensitizer to the **TA** substrate, associated with the low driving force of the process. For this reason, we envisioned that replacing the ruthenium sensitizer with cyclometalated iridium complexes **Ir1** and **Ir2** could improve the photoelectrochemical performances by taking advantage of a more oxidizing power (*E* = +1.42 and +1.68 V vs SCE for **Ir1** and **Ir2**, respectively, vs *E* = +1.26 V for the ruthenium sensitizer).^20^ Starting from this background, we thus tested our TiO_2_ electrodes sensitized with both **Ir1** and **Ir2** to promote the Diels–Alder reaction between **TA** and **ISO**. To this purpose, we employed TiO_2_ electrodes featuring both the triple layer and the scattering layer that proved successful in the TEMPO-mediated BzOH oxidation. Concomitantly, we adopted the solution conditions optimized by Schanze and coworkers,^19^ namely [**TA**] = 50 mM, [**ISO**] = 150 mM, and 0.1 M LiTFSI as a supporting electrolyte in acetonitrile.

Figure 4a depicts the JV curves obtained for both **Ir1**- and **Ir2**-sensitized electrodes. Inspection of the curves shows similar voltammetric profiles for both electrode types with onset potentials of ca. −0.4 V vs SCE and comparable current densities at plateau in the order of ca. 0.7 mA·cm^–2^, with slightly improved photocurrents and a more squared profile in the case of **Ir2**. The recorded values are considerably larger than those measured for TiO_2_ electrodes sensitized with the ruthenium polypyridine dye (0.15 mA·cm^–2^),^19^ immediately highlighting the benefits attained by the use of strongly oxidizing iridium complexes. Interestingly, the JV curves under chopped irradiation display minor spikes only in the case of complex **Ir1** and at potentials below 0 V vs SCE, pointing out reduced recombination routes in the presence of the **TA/ISO** combination with respect to the TEMPO-mediated system previously described.

**Figure 4:**
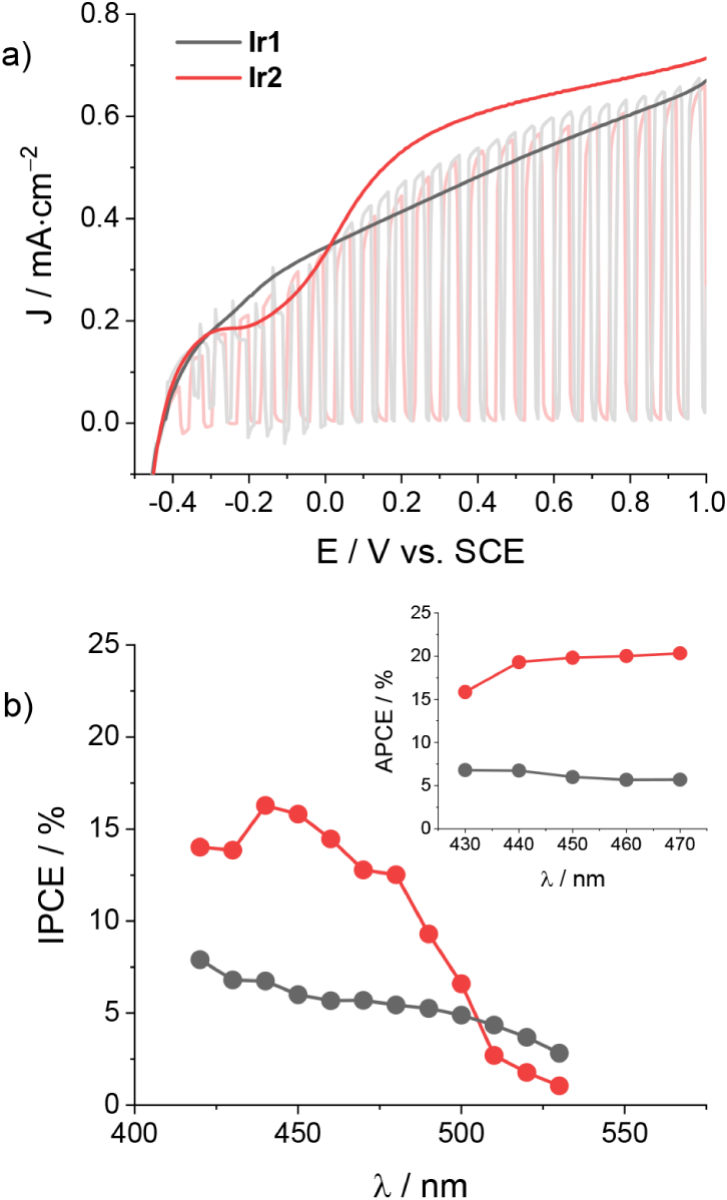
a) JV curves of TiO_2_-sensitized electrodes (triple layer + scattering layer) with **Ir1** and **Ir2** under direct or chopped irradiation (1 sun, cutoff filter at 395 nm) and b) the corresponding IPCE spectra and APCE spectra (inset) at +0.5 V vs SCE in the presence of 50 mM **TA**, 150 mM **ISO**, and 0.1 M LiTFSI in acetonitrile.

This experimental evidence well complies with the results obtained from inspection of the photoaction spectra (Figure 4b), where the IPCE reaches maximum values of 8% and 16% in the case of **Ir1** and **Ir2**, respectively. Normalization of the IPCE by the LHE of the photoelectrodes finally yields appreciably straight lines in the 430–480 nm region (inset in Figure 4b) providing average APCE values of 6% and 19% for **Ir1** and **Ir2**, respectively. Overall, these results support enhanced charge collection efficiencies for both TiO_2_ electrodes when tested in the Diels–Alder reaction, compared to the TEMPO-mediated oxidation of BzOH. Notably, the electrode sensitized with the fluorinated **Ir2** complex showed superior efficiency, likely as the result of improved charge-transfer kinetics at the electrode–solution interface and more effective suppression of charge recombination between the oxidized electrolyte and the injected electron in the TiO_2_ conduction band, as previously envisioned.

We finally turned to monitoring the long-term performances of our sensitized electrodes by performing bulk electrolysis experiments upon application of a constant potential of +0.5 V vs SCE. The photoreaction was monitored over a time frame of 2 h until a noticeable abatement of the photocurrent density was apparent (Figure S9). After this time, we evaluated (Figures S10 and S11) both the consumption of **TA** and the formation of the 4-(*p*-methoxyphenyl)-1,5-dimethylcyclohexene Diels–Alder product (**1**). An almost quantitative conversion of TA is registered (92% and 99% for **Ir1** and **Ir2**, respectively), while the coupling product **1** is formed with decent yields of 62% and 64% for **Ir1** and **Ir2**, respectively. Although we do not detect the parallel formation of a [2 + 2] cycloaddition product as previously reported,^19^ the failure to observe quantitative yields might suggest the occurrence of minor side reactions involving the **TA** radical cation. This issue could be, in principle, minimized by working under a large excess of **ISO**;^19^ however, optimization of the product yield is out of the scope of the present work. FEs of 613% and 945% for **Ir1** and **Ir2**, respectively, were finally calculated, considering one electron per product molecule, whose values exceeding unity can be anticipated according to the chain mechanism of the reaction here involved.^19,46,47^

Though visual inspection of the post-electrolysis electrodes indicates some dye desorption for both **Ir1**- and **Ir2**-sensitized TiO_2_ electrodes after 2 h electrolysis, the leveling off of the photocurrent densities within the time frame of 2 h can be mainly accounted for by considering the progressive depletion of the **TA** substrate. Thus, the more pronounced abatement in photocurrent density over time (Figure S9) and the enhanced FEs obtained with **Ir2** with respect to **Ir1** nicely match the improved quantum efficiency observed in the case of the fluorinated complex.

### Transient Absorption Spectroscopy

Regeneration and recombination dynamics were probed by means of transient absorption spectroscopy (TAS) in the μs-ms time scale on dyed TiO_2_ thin films. The TA spectra upon 355 nm excitation of **Ir1** and **Ir2** electrodes in contact with a 0.1 M LiTFSI/acetonitrile solution are depicted in Figure 5, covering the 380–800 nm spectral window.

**Figure 5:**
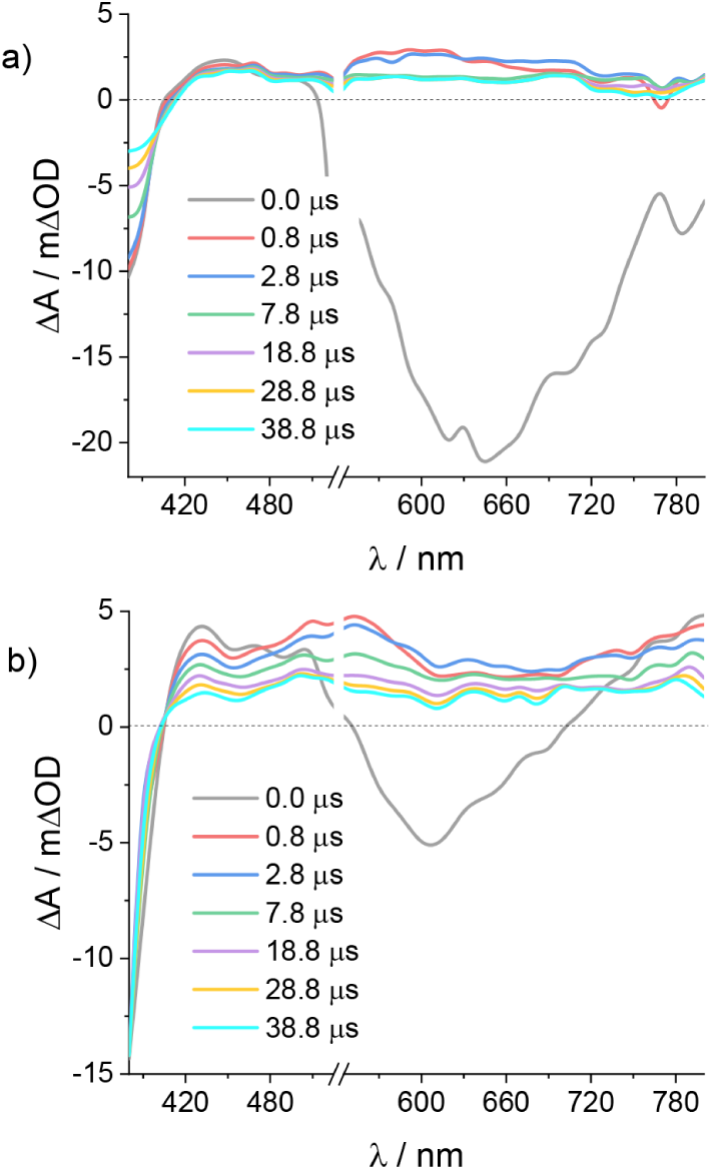
Transient absorption spectra recorded for TiO_2_ electrode sensitized with a) **Ir1** and b) **Ir2** in contact with 0.1 M LiTFSI/ACN solution upon 355 nm excitation. Input impedance: 350 Ω.

Consistent with the nonunitary charge injection efficiencies η_inj_) evaluated in the presence of LiI (78% for **Ir1** and 74% for **Ir2**), the early time TA spectra clearly revealed a residual population of the lowest triplet excited state (T_1_). This state is characterized by a bleaching feature mirroring the ground state absorption manifold for λ < 400 nm, a T_1_ → T_*n*_ absorption band between 410 and 510 nm, and an intense spontaneous emission for λ > 510 nm. Consistent with the stationary emission spectra reported in Figure 1a, the emission of **Ir2** is blue-shifted relative to **Ir1**. The T_1_ residue may decay either via radiative pathways, as suggested by the strong emission, or via electron injection. Such dynamics are essentially complete within 800 ns, after which the difference spectra are solely dominated by the charge-separated state (Ir­(IV)/TiO_2_(e^–^)). This is defined by a bleached MLCT band below 400 nm and a weak, featureless absorption extending into the red region, likely originating from LMCT transitions. From 700 nm onward, electrons photoinjected and trapped in TiO_2_ contribute to the flat absorption.^48^

Since these spectral features persist for tens of ms, recombination dynamics were further probed by monitoring the kinetics at 450 nm for both **Ir1** and **Ir2**, extending the time window to 50 ms (Figure S12). To optimize the signal-to-noise ratio (S/N), oscillographic traces were averaged over 100 shots and preamplified by using input impedances of 350 Ω, 10 kΩ or 1 MΩ. The recombination dynamics were modeled by a combination of a power law function (eq 1) and a Kohlrausch–Williams–Watts (KWW) stretched-exponential function (eq 2). The power law describes the fast bimolecular recombination of photogenerated electron–hole pairs, accounting for ca. 50% of the amplitude recovery within the first 50 μs. The KWW captures the intrinsic heterogeneity of the mesoporous surface, which generates a distribution of recombination rates due to electron trapping and detrapping near the semiconductor conduction band. The stretching parameter β in the KWW was set to 0.25 for both complexes.

1 $$\Delta A_{t<50\mu s}=A+bt^{-c}$$

$$\Delta A_{t>50\mu s}=A^{\prime }+b^{\prime }e^{{-(\frac{t}{\tau_{0}})}^{\beta }}$$ 2

3 $$\left\langle \tau \right\rangle =\frac{\tau_{0}}{\beta }\Gamma \left(\frac{1}{\beta }\right)$$

Weighted lifetimes were then computed from τ_0_ according to eq 3, where Γ denotes the gamma function.^49^ The resulting <τ> were 4.3 ms for **Ir1** and 1.1 ms for **Ir2**, corresponding to recombination rate constants (*k*_rec_) on the order of 10^3^–10^4^ s^–1^. Importantly, under no circumstances are such small differences in lifetime relevant for DSPECs operations, since upon addition of either 10 mM of TEMPO or 50 mM of **TA** to the electrolyte, the pseudo-first-order rate constant associated with regeneration (*k*_reg_) reached values on the order of 10^6^ s^–1^ (Figure S12). Since *k*_reg_ ≫ *k*_rec_, the regeneration efficiency (η_reg_, eq 4) approaches unity.

4 $$\eta_{reg}=\frac{k_{reg}}{k_{reg}+k_{rec}}$$

## Discussion

A detailed inspection of the experimental results obtained with the cyclometalated iridium complexes **Ir1** and **Ir2** reveals important insights into the use of this class of sensitizers toward light-driven organic transformations on sensitized TiO_2_ photoanodes. In this regard, the quantum efficiency has been shown to depend on the combination of the nature of the sensitizer and the target reaction. APCE values of 2.4% and 2.2% were indeed determined in the TEMPO-mediated oxidation process for **Ir1** and **Ir2**, respectively, whereas larger values of 6% and 19% were obtained in the Diels–Alder reaction for **Ir1** and **Ir2**, respectively. The APCE can be expressed according to eq 5, where η_inj_ is the efficiency of the electron injection into TiO_2_ and η_cc_ is the charge collection efficiency. This latter can be further expanded according to eq 6, where η_reg_ represents the efficiency of dye regeneration by the electrolyte and η_rec_ the efficiency of the charge recombination between the oxidized electrolyte and the injected electron.

5 $$APCE=IPCE\left(\lambda \right)/LHE=\eta_{inj}\cdot \eta_{cc}$$

6 $$\eta_{cc}=\eta_{reg}\cdot \left(1-\eta_{rec}\right)$$

The photoelectrochemical experiments conducted in the presence of LiI as a sacrificial hole scavenger (see above) indicate efficient charge injection (η_inj_ of 78% and 74% for **Ir1** and **Ir2**, respectively), in agreement with evidence from ultrafast spectroscopy studies on TiO_2_ thin-films sensitized with iridium complexes.^41^ However, the charge injection efficiency (η_inj_) is not expected to differ considerably for the two different organic reactions, considering that the same solvent and supporting electrolyte have been employed. Thus, the main factors influencing the overall quantum efficiency in the target organic transformations are dye regeneration (η_reg_) and charge recombination (η_rec_).

Under these assumptions, the modest APCE measured for both iridium complexes in the TEMPO-mediated BzOH oxidation very likely originates from favorable recombination processes involving the oxidized TEMPO and the injected electron. This is not fully unexpected considering the reversible nature of the redox mediator.^50^ In this regard, the similar values observed for both iridium complexes nicely align with this hypothesis. Thus, the driving force advantage expected in the case of the fluorinated complex **Ir2** turns out to be practically irrelevant.

On the other hand, the larger APCEs measured for both **Ir1** and **Ir2** in the Diels–Alder reaction suggest a partial suppression of detrimental charge recombination channels, consistent with the expectedly irreversible behavior due to the reactivity of the oxidized **TA** intermediate in the presence of an excess of **ISO**. Within this context, the appreciable enhancement observed when moving from **Ir1** to **Ir2** can be ascribed to a more effective charge transfer process to the **TA** substrate, thanks to the larger driving force available, as well as to the packing of **Ir2** on top of TiO_2_, originating from the presence of fluorine groups, likely limiting permeation of the oxidized **TA** toward the TiO_2_ surface. These findings nicely corroborate the observation of large APCEs in DSPEC employing perfluorinated porphyrin sensitizers.^14^ Furthermore, the use of fluorinated monolayers was also shown to effectively suppress charge recombination phenomena in DSSCs.^51^

Photoelectrochemical tests under long-term operation show similarly that the gain expected using the fluorinated **Ir2** complexes against the unsubstituted **Ir1** dye is attained only in the case of the Diels–Alder reaction, i.e., under conditions where no redox mediator is involved to power the desired organic transformation. In fact, when the iridium complexes are employed in the presence of a reversible redox mediator such as the TEMPO radical the photoelectrochemical performance substantially drops for both iridium complexes, with a stronger effect in the case of the fluorinated complex **Ir2**. This can be attributed to the local depletion of the TEMPO radical, associated with the rate-determining catalytic step between the oxidized TEMPO^+^ and the BzOH substrate, possibly decreasing the regeneration yield during bulk electrolysis conditions. This would lead to the accumulation of oxidized iridium­(IV) species at the electrode surface, from which degradation phenomena can become competitive. The inherent instability of iridium­(IV) species is indeed not unexpected, considering their strong reactivity, likely implying simultaneous oxidation of the ligand.^52^ Under these conditions, it is highly plausible that the more positive oxidation potential of the fluorinated **Ir2** complex, compared to the unsubstituted **Ir1**, enhances the susceptibility of the corresponding Ir­(IV) species toward self-degradation, thereby explaining the lower FE of the TiO_2_ electrode sensitized with the fluorinated **Ir2** complex toward benzaldehyde formation. Thus, the sought increase in the oxidative power of the oxidized sensitizer, expected to improve the performance of the DSPEC based on simple thermodynamic grounds, while effective in the Diels–Alder reaction, turns out to be counterproductive when the organic transformation involves a redox mediator.

## Conclusion

In this study, we have reported for the first time the use of cyclometalated iridium complexes as photosensitizers anchored onto mesoporous TiO_2_ within DSPEC configurations for the generation of value-added organic compounds. Specifically, we investigated two sensitizers, **Ir1** and **Ir2**, which differ in the presence of fluorinated substituents that enhance their oxidative power toward organic substrates. Our findings demonstrate that these iridium complexes effectively promote both the TEMPO-mediated oxidation of BzOH and the radical cation Diels–Alder reaction between TA and ISO, yielding the coupling product **1**. However, the TEMPO-mediated oxidation of BzOH to benzaldehyde exhibits reduced efficiency, primarily due to degradation processes, which are more pronounced in the case of the fluorinated complex. In contrast, significantly improved performance is observed in the DSPEC system that operates without a redox mediator, namely in the Diels–Alder reaction. Under these conditions, the increased oxidative strength of the sensitizer proves also advantageous, leading to enhanced reactivity.

These findings underscore that the performance of a photosensitizer in a DSPEC system is highly dependent on the specific target reaction. Therefore, the selection of the dye component in DSPECs should be strategically tailored to the desired transformation. We believe this work offers valuable insights into the design and application of highly oxidizing iridium complexes and potentially other coordination compounds for the photoelectrochemical activation of organic substrates.

## Supplementary Material



## Notes

Additional photophysical and electrochemical data, additional photoelectrochemical characterization, Faradaic yield determination, and laser flash photolysis studies (PDF)
