# Supplementary material for: Exploring the Boundaries of Cyclometalated Iridium(III) Sensitizers in Photoelectrochemical Organic Transformations
Source: ACS Appl Mater Interfaces. 2025 Dec 12;17(51):69519–29. doi: 10.1021/acsami.5c20212 (PMC12754757; doi:10.1021/acsami.5c20212)
Supplement: Supplementary file 1 [file am5c20212_si_001.pdf]

**Supporting Information**

# Exploring the Boundaries of Cyclometallated Iridium(III) Sensitizers in Photoelectrochemical Organic Transformations

*Andrea Mantovani,*[‡] *Annagioia Mastrolorenzo,*[†] *Edoardo Marchini,*[‡]* *Paola Manini,* [†]*

*and Mirco Natali*[‡]*

[‡] Department of Chemical, Pharmaceutical and Agricultural Sciences (DOCPAS), University of Ferrara, Via L. Borsari 46, 44121 Ferrara, Italy.

E-mail: edoardo.marchini@unife.it; mirco.natali@unife.it

[†] Department of Chemical Sciences, University of Naples Federico II, via Cintia 4, I-80126 Napoli, Italy.

E-mail: pmanini@unina.it

## Table of Content

**S1. Photophysical characterization** p. S2

**S2. Electrochemical characterization** p. S5

**S3. TEMPO-mediated BzOH oxidation** p. S6

**S4. Radical cation Diels-Alder reaction** p. S12

**S5. Transient absorption spectroscopy** p. S16

**S6. References of the Supporting Information** p. S17

## S1. Photophysical characterization

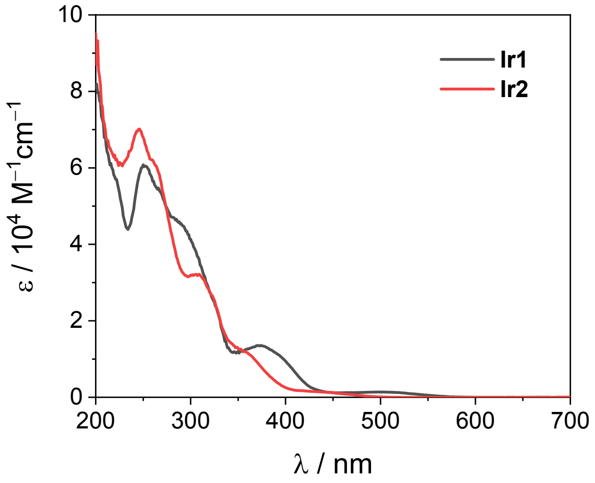


a)

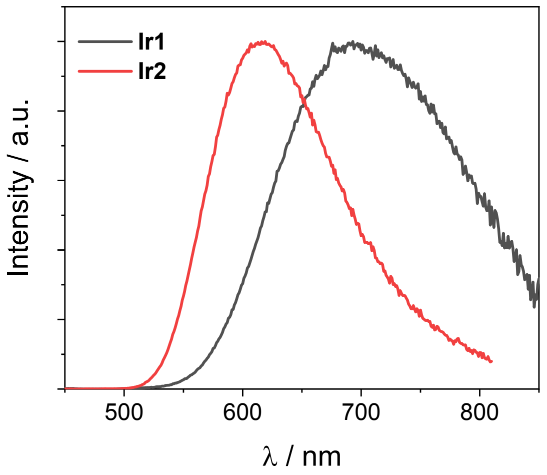


b)

**Figure S1**. a) Absorption and b) emission spectra (excitation at 400 nm) of **Ir1** and **Ir2** in diluted acetonitrile solution.

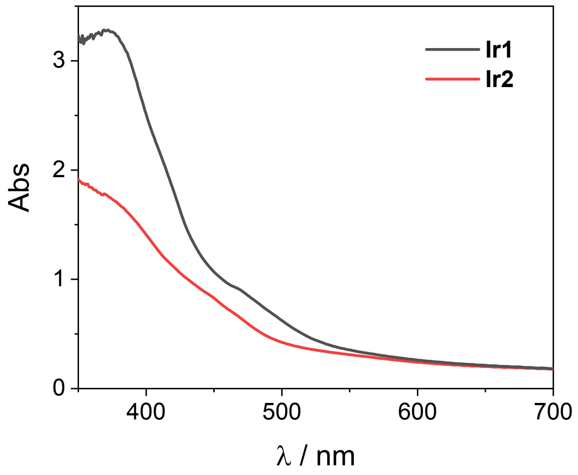


a)

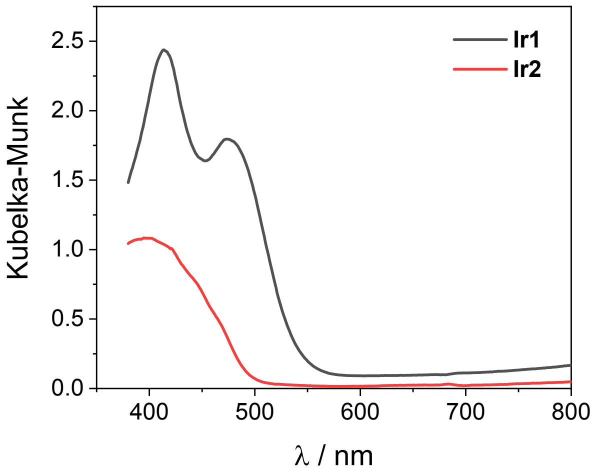


b)

**Figure S2**. Absorption spectra of **Ir1** and **Ir2** on $TiO_2$ electrodes: a) triple layer (recorded in trasmittance mode) and b) triple layer + scattering layer (recorded in diffuse reflectance mode).

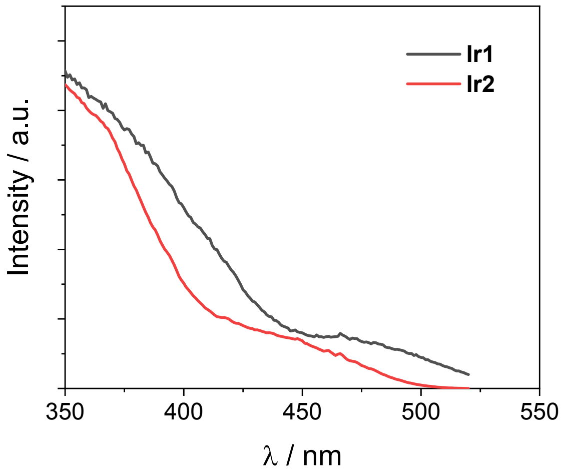


**Figure S3**. Excitation spectra of **Ir1** (emission recorded at 615 nm) and **Ir2** (emission recorded at 550 nm) on $ZrO_2$ electrodes.

**Table S1**. Summary of photophysical data.

| **Compound** | **Acetonitrile solution** | | | | **$ZrO_2$** |
|---|---|---|---|---|---|
| | UV-vis $\lambda_{max}$ / nm ($\varepsilon$ / $M^{-1}$ $cm^{-1}$) | PL $\lambda_{em}$ / nm | $\Phi$ / % [a] | $\tau$ / ns [b] | PL $\lambda_{em}$ / nm |
| **Ir1** | 250 (60700), 370 (sh), 491 (sh) | 693 | 3.4 | 42 | 614 |
| **Ir2** | 245 (70223), 263 (sh) 315 (sh), 363 (sh), 443 (sh) | 616 | 30.5 | 810 | 553 |

[a] determined using $[Ru(bpy)_3]^{2+}$ as a standard ($\Phi$ = 0.062 in ACN solution); [b] excitation at 355 nm.

## S2. Electrochemical characterization

**Table S2**. Summary of relevant electrochemical data.

| Compound | $E_{Ox}$ / V vs SCE | $E_{Red}$ / V vs SCE | $E_{Ox}$* / V vs SCE |
|---|---|---|---|
| **Ir1** | +1.42 | −1.37 | −0.88 |
| **Ir2** | +1.69 | −1.28 | −0.80 |
| **TEMPO** | +0.66 [a] | | |
| **TA** | +1.11 [b] | | |
| **$TiO_2$(CB)** | | −0.70 [c] | |

[a] Taken from ref. S1; [b] taken from ref. S2; [c] taken from ref. S3.

## S3. TEMPO-mediated BzOH oxidation

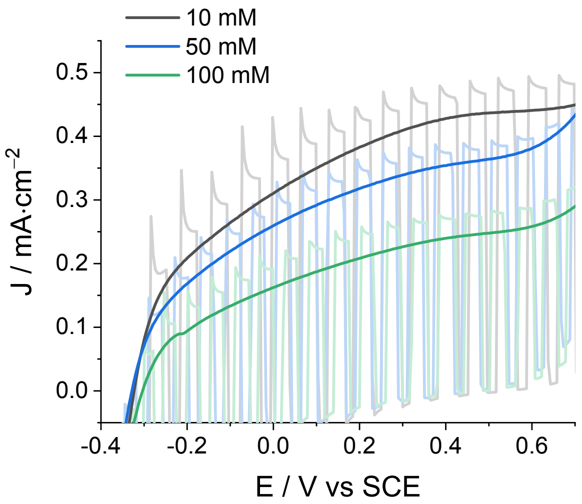


a)

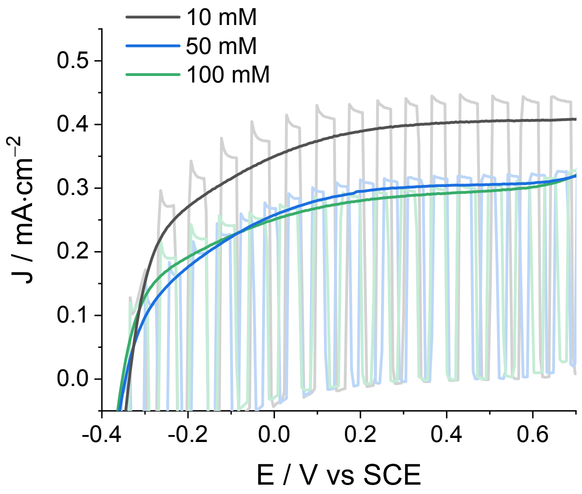


b)

**Figure S4**. JV curves under direct or chopped irradiation (1 sun, cut-off filter at 395 nm) of $TiO_2$-sensitized electrodes (triple layer) for a) **Ir1** and b) **Ir2** at variable TEMPO concentration (10-100 mM) in acetonitrile solution (0.1 M $LiClO_4$).

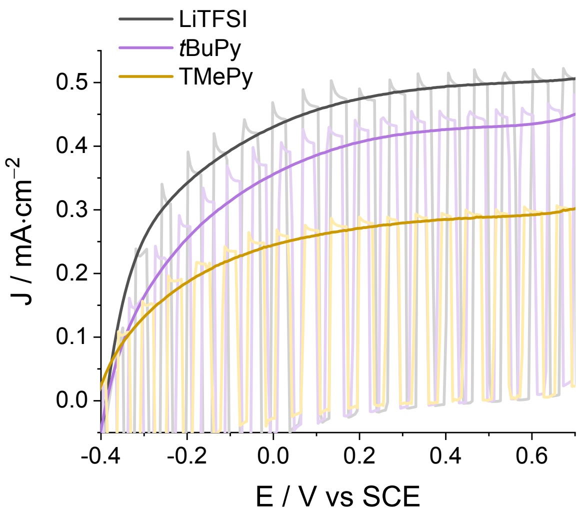


a)

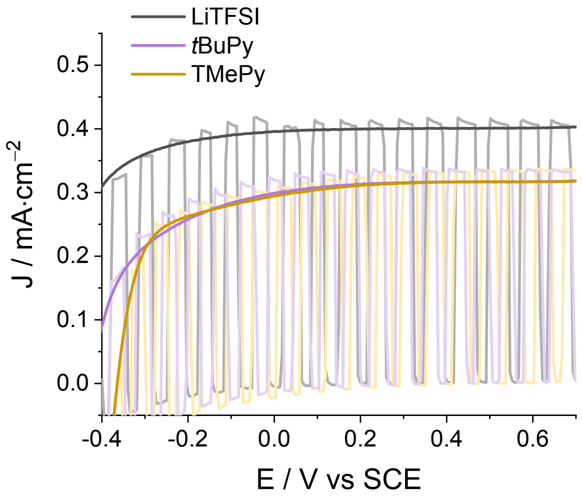


b)

**Figure S5**. JV curves under direct or chopped irradiation (1 sun, cut-off filter at 395 nm) of $TiO_2$-sensitized electrodes (triple layer) for a) **Ir1** and b) **Ir2** at 10 mM TEMPO in acetonitrile solution in the presence of 0.1 M LiTFSI, 0.1 M *t*BuPy (with 0.1 M $LiClO_4$) and 0.1 M TMPy (with 0.1 M $LiClO_4$). LiTFSI = lithium bis(trifluoromethansulfonylimide), *t*BuPy = 4-*tert*-butylpyridine, TMePy = 2,4,6-trimethylpyridine.

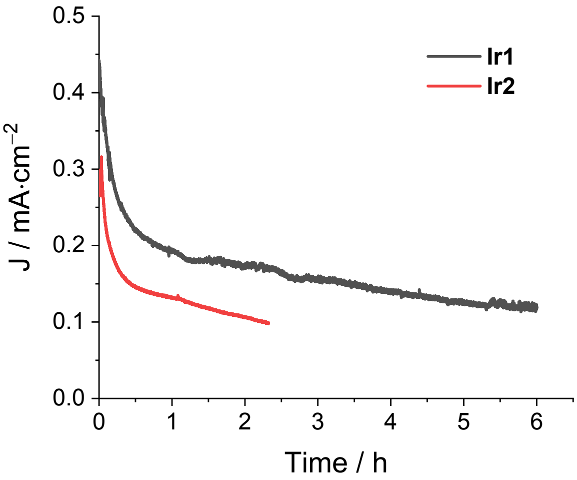


**Figure S6**. Chronoamperometric curves under direct irradiation (1 sun, cut-off filter at 395 nm) at +0.5 V vs. SCE of $TiO_2$-sensitized electrodes (triple layer + scattering layer) for **Ir1** and **Ir2** at 10 mM TEMPO in acetonitrile solution in the presence of 0.1 M LiTFSI and 50 mM BzOH.

The amount of benzaldehyde formed after bulk electrolysis was estimated by $^1$H-NMR using DMF as an internal standard by comparing the intensity of the peaks of the benzaldehyde at δ = 9.8 ppm (1 H), δ = 7.45 ppm (2 H), and δ = 7.3 ppm (3 H) with the signal at δ = 2.9 ppm (3 H) of the DMF standard. The Faradaic efficiency (FE) was determined considering the total charge passed (obtained by integration of the chronoamperometric traces in Figure S6) and accounting for the 2-electron nature of the oxidation process.

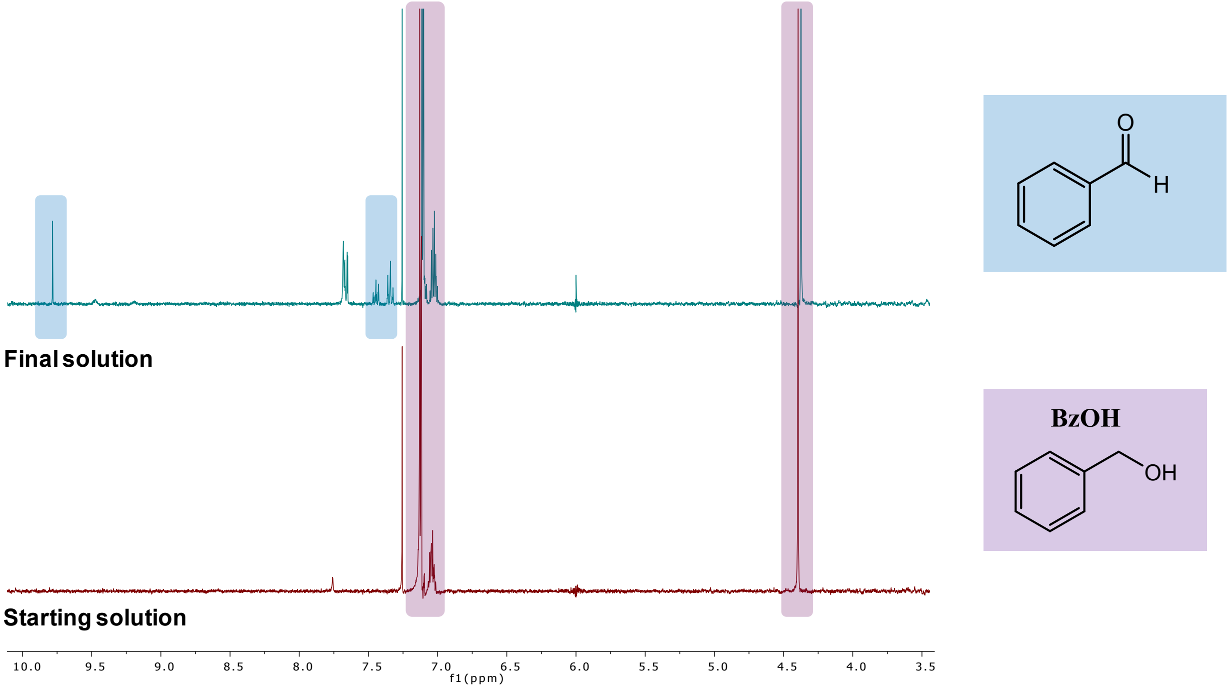


**Figure S7**. $^1$H-NMR analysis in $CDCl_3$ before and after the bulk electrolysis (6 h) at +0.5 V vs SCE of $TiO_2$ electrodes (triple layer + scattering layer) sensitized with **Ir1** at 10 mM TEMPO in acetonitrile solution in the presence of 0.1 M LiTFSI and 50 mM BzOH.

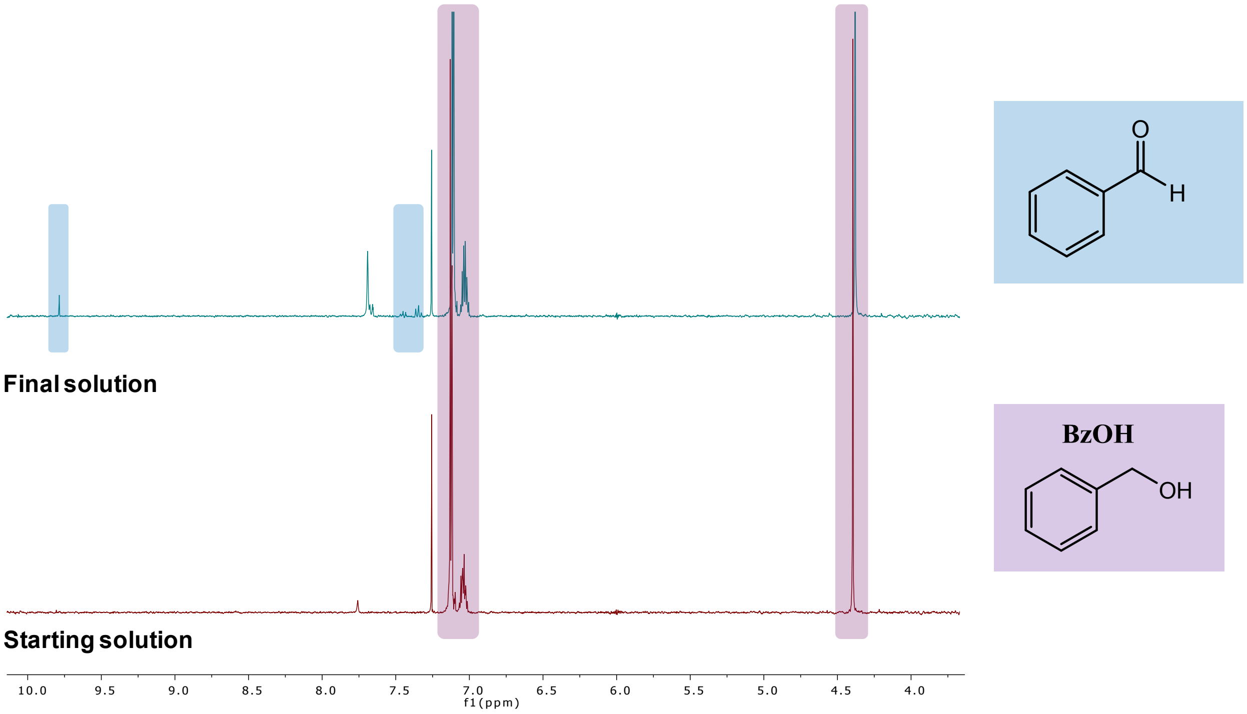


**Figure S8**. $^{1}$H-NMR analysis in $CDCl_3$ before and after the bulk electrolysis (2 h) at +0.5 V vs SCE of $TiO_2$ electrodes (triple layer + scattering layer) sensitized with **Ir2** at 10 mM TEMPO in acetonitrile solution in the presence of 0.1 M LiTFSI and 50 mM BzOH.

**Table S3**. Comparison of DSPECs for TEMPO-mediated BzOH oxidation to benzaldehyde.

| **Conditions** | **J / μA·cm$^{-2}$** | **IPCE / %** | **FE / %** | **Ref** |
|---|---|---|---|---|
| *Nano*ITO, Ru-TEMPO dyad, $H_2O$, pH 10 | <180 | n.d. | 80 | S4 |
| $TiO_2$, ZnP-TEMPO dyad, $CH_3CN$ | 200 | 2.6 | 82 | S5 |
| $TiO_2$, organic dye, $CH_3CN$ | 400 | n.d. | 100 | S6 |
| $TiO_2$, DPP, $H_2O$, pH 8 | 90 | 2 | 87 | S7 |
| ITO, perylene, $CH_3CN$ | 18 | n.d. | n.d. | S8 |
| $SnO_2/TiO_2$, ZnP (BETA-4), $CH_3CN$ | 750 | 18 | 100 | S9 |
| $TiO_2$, **Ir1**, $CH_3CN$ | 670 | 5.5 | 42 | This work |
| $TiO_2$, **Ir2**, $CH_3CN$ | 510 | 1.9 | 32 | This work |

n.d. = not determined

## S4. Radical cation Diels-Alder reaction

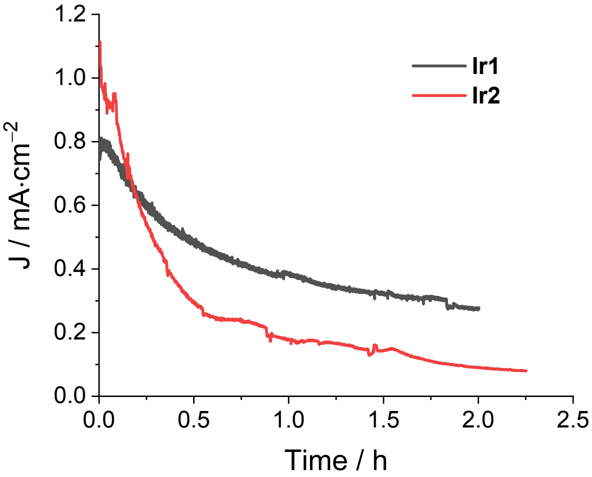


**Figure S9**. Chronoamperometric curves under direct irradiation (1 sun, cut-off filter at 395 nm) at +0.5 V vs. SCE of $TiO_2$-sensitized electrodes (triple layer + scattering layer) for **Ir1** and **Ir2** at 50 mM **TA**, 150 mM **ISO** in acetonitrile solution in the presence of 0.1 M LiTFSI.

The amount of **1** formed after bulk electrolysis was estimated by $^{1}$H-NMR using DMF as an internal standard by comparing the intensity of the peaks at δ = 5.2 ppm (1 H), δ = 6.65 ppm (2 H), and δ = 6.95 ppm (2 H) with the signal at δ = 7.8 ppm (1 H) of the DMF standard. The Faradaic efficiency (FE) was determined considering the total charge passed (obtained by integration of the chronoamperometric traces in Figure S9) and assuming one electron transfer process. The amount of **TA** consumed was estimated similarly by integration of the peak at δ = 7.05 ppm (2 H), if observed.

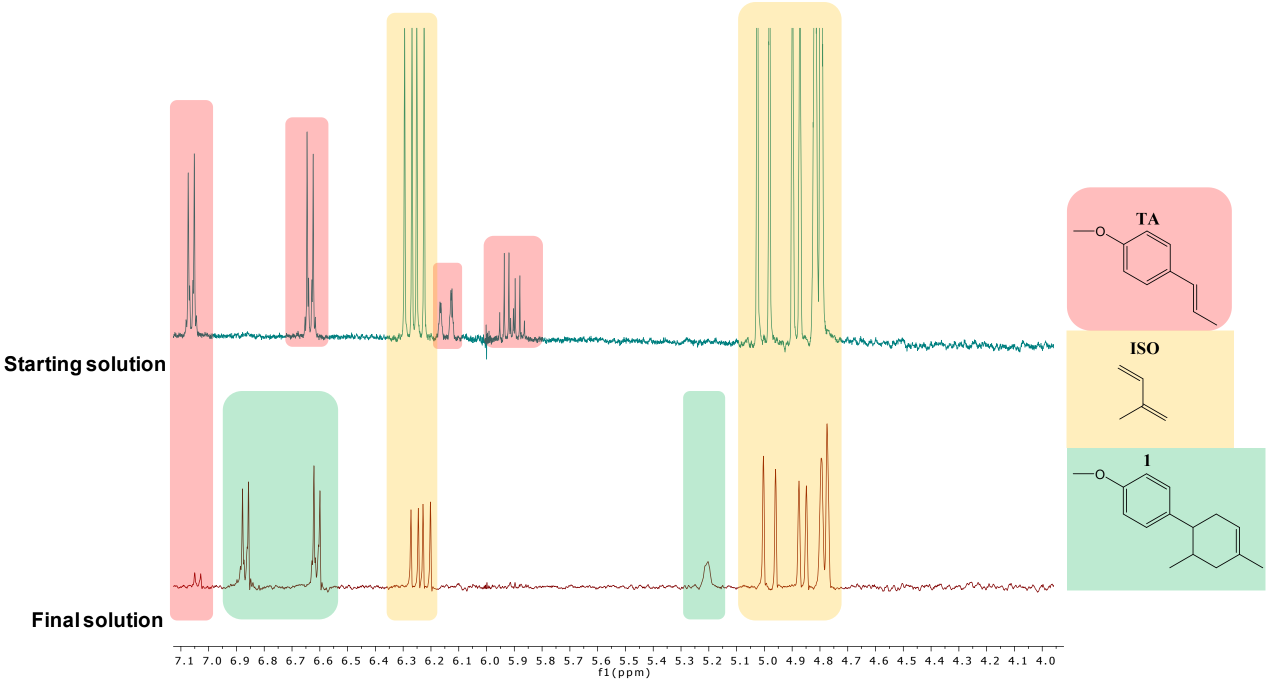


**Figure S10**. $^{1}$H-NMR analysis in $CDCl_3$ before and after the bulk electrolysis (2 h) at +0.5 V vs SCE of $TiO_2$ electrodes (triple layer + scattering layer) sensitized with **Ir1** at 50 mM **TA**, 150 mM **ISO** in acetonitrile solution in the presence of 0.1 M LiTFSI.

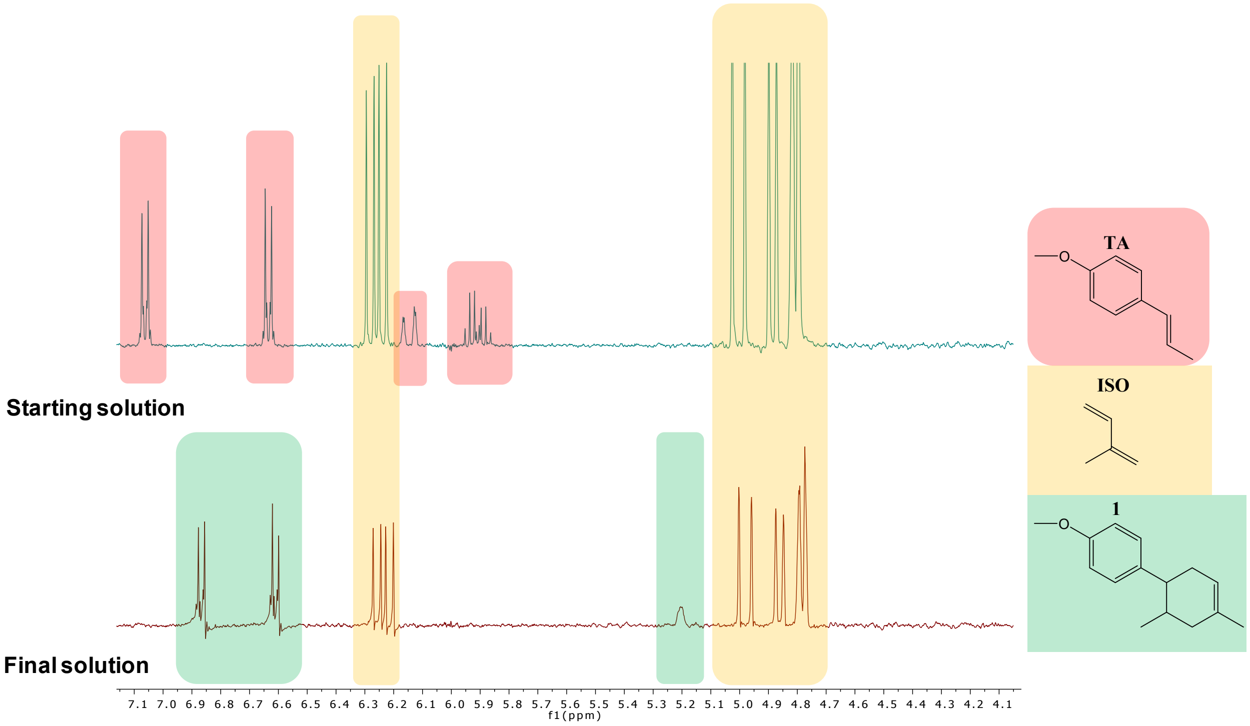


**Figure S11**. $^1$H-NMR analysis in $CDCl_3$ before and after the bulk electrolysis (2 h) at +0.5 V vs SCE of $TiO_2$ electrodes (triple layer + scattering layer) sensitized with **Ir2** at 50 mM **TA**, 150 mM **ISO** in acetonitrile solution in the presence of 0.1 M LiTFSI.

**Table S4**. Comparison of DSPECs for photoelectrochemical Diels-Alder.

| Conditions | $J$ / µA·cm$^{-2}$ | IPCE / % | Ref |
|---|---|---|---|
| $TiO_2$, RuP, $CH_3CN$ | 150 | 3 [a] | S2 |
| $TiO_2$, **Ir1**, $CH_3CN$ | 600 | 8 | This work |
| $TiO_2$, **Ir2**, $CH_3CN$ | 650 | 16 | This work |

[a] Estimated from the APCE and the LHE according to APCE = IPCE / LHE.

## S5. Transient absorption spectroscopy

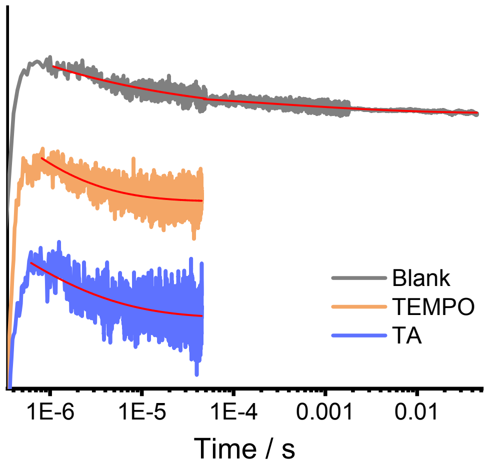


a)

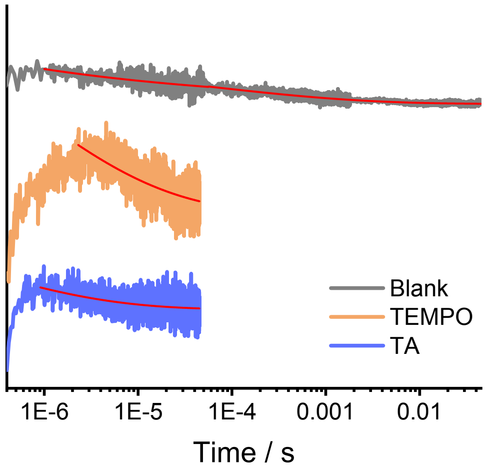


b)

**Figure S12.** Kinetic traces at 450 nm obtained by laser flash photolysis (excitation at 355 nm) for a) **Ir1** and b) **Ir2** in contact with the blank electrolyte (0.1 M LiTFSI), and with the target substrates: TEMPO (10 mM TEMPO, 0.1 M LiTFSI) or **TA** (50 mM TA, 0.1 M LiTFSI).

## S6. References of the Supporting Information


S1 Zhang, Z.; Chen, P.; Murakami, T. N.; Zakeeruddin, S. M., Grätzel, M. The 2,2,6,6-Tetramethyl-1-piperidinyloxy Radical: An Efficient, Iodine-Free Redox Mediator for Dye-Sensitized Solar Cells. *Adv. Funct. Mater.* **2008**, *18*, 341-346.

S2 Turlington, M. D.; Ahmed, S.; Schanze, K. S. Radical Cation Diels−Alder Reaction by Photocatalysis at a Dye Sensitized Photoanode. *ACS Catal.* **2024**, *14*, 12512-12517.

S3 Hagfeldt, A.; Boschloo, G.; Sun, L.; Loo, L.; Pettersson, H. Dye-Sensitized Solar Cells. *Chem. Rev.* **2010**, *110*, 6595-6663.

S4 Pati, P. B.; Abdellah, M.; Diring, S.; Hammarström, L.; Odobel, F. Molecular Triad Containing a TEMPO Catalyst Grafted on Mesoporous Indium Tin Oxide as a Photoelectrocatalytic Anode for Visible Light-Driven Alcohol Oxidation. *ChemSusChem* **2021**, *14*, 2902-2913.

S5 Nikolaudakis, E.; Pati, P. B.; Charalambidis, G.; Budkina, D. S.; Diring, S.; Planchat, A.; Jacquemin, D.; Vauthey, E.; Coutsolelos, A. G.; Odobel, F. Dye-Sensitized Photoelectrosynthesis Cells for Benzyl Alcohol Oxidation Using a Zinc Porphyrin Sensitizer and TEMPO Catalyst. *ACS Catal.* **2021**, *11*, 12075-12086.

S6 Bruggeman, D. F.; Mathew, S.; Detz, R. J.; Reek, J. N. H. Comparison of Homogeneous and Heterogeneous Catalysts in Dye-Sensitised Photoelectrochemical Cells for Alcohol Oxidation Coupled to Dihydrogen Formation. *Sustain. Energy Fuels* **2021**, *5*, 5707-5716.

S7 Antón-Garcia, D.; Moore, E. E.; Bajada, M. A.; Eisenschmidt, A.; Oliveira, A. R.; Pereira, I. A. C.; Warnan, J.; Reisner, E. Photoelectrochemical Hybrid Cell for Unbiased $CO_2$ Reduction Coupled to Alcohol Oxidation. *Nat. Synth.* **2022**, *1*, 77-86.

S8 Zhuang, J. L., Shen, Y. M.; Xue, Y.; Yan, M.; Cheng, H.; Chen, Z.; Yu, X. J.; Lian, X. B.; Zhu, S. B. Electrochemical Deposition of Perylene-Based Thin Films from Aqueous Solution and Studies of Visible-Light-Driven Oxidation of Alcohols. *ACS Appl. Energy Mater.* **2020**, *3*, 9098-9106.

S9 Di Carlo, G.; Albanese, C.; Molinari, A.; Carli, S.; Argazzi, R.; Minguzzi, A.; Tessore, F.; Marchini, E.; Caramori, S. Perfluorinated Zinc Porphyrin Sensitized Photoelectrosynthetic Cells for Enhanced TEMPO-Mediated Benzyl Alcohol Oxidation. *ACS App. Mater. Interfaces* **2024**, *16*, 14864-14882.
